# Interpretable Machine Learning for Characterization of Focal Liver Lesions by Contrast-Enhanced Ultrasound

**DOI:** 10.1109/TUFFC.2022.3161719

**Published:** 2022-04-27

**Authors:** Simona Turco, Thodsawit Tiyarattanachai, Kambez Ebrahimkheil, John Eisenbrey, Aya Kamaya, Massimo Mischi, Andrej Lyshchik, Ahmed El Kaffas

**Affiliations:** Department of Electrical Engineering, Eindhoven University of Technology, 5612 AZ Eindhoven, The Netherlands; Department of Radiology, Stanford Medicine, Stanford, CA 94305 USA.; Department of Electrical Engineering, Eindhoven University of Technology, 5612 AZ Eindhoven, The Netherlands; Department of Radiology, Thomas Jefferson University, Philadelphia, PA 19107 USA.; Department of Radiology, Stanford Medicine, Stanford, CA 94305 USA.; Department of Electrical Engineering, Eindhoven University of Technology, 5612 AZ Eindhoven, The Netherlands; Department of Radiology, Thomas Jefferson University, Philadelphia, PA 19107 USA.; Department of Radiology, Stanford Medicine, Stanford, CA 94305 USA.

**Keywords:** Medical imaging, medical signal and image processing, medical tissue characterization, ultrasound (US) contrast agents

## Abstract

This work proposes an interpretable radiomics approach to differentiate between malignant and benign focal liver lesions (FLLs) on contrast-enhanced ultrasound (CEUS). Although CEUS has shown promise for differential FLLs diagnosis, current clinical assessment is performed only by qualitative analysis of the contrast enhancement patterns. Quantitative analysis is often hampered by the unavoidable presence of motion artifacts and by the complex, spatiotemporal nature of liver contrast enhancement, consisting of multiple, overlapping vascular phases. To fully exploit the wealth of information in CEUS, while coping with these challenges, here we propose combining features extracted by the temporal and spatiotemporal analysis in the arterial phase enhancement with spatial features extracted by texture analysis at different time points. Using the extracted features as input, several machine learning classifiers are optimized to achieve semiautomatic FLLs characterization, for which there is no need for motion compensation and the only manual input required is the location of a suspicious lesion. Clinical validation on 87 FLLs from 72 patients at risk for hepatocellular carcinoma (HCC) showed promising performance, achieving a balanced accuracy of 0.84 in the distinction between benign and malignant lesions. Analysis of feature relevance demonstrates that a combination of spatiotemporal and texture features is needed to achieve the best performance. Interpretation of the most relevant features suggests that aspects related to microvascular perfusion and the microvascular architecture, together with the spatial enhancement characteristics at wash-in and peak enhancement, are important to aid the accurate characterization of FLLs.

## I. INTRODUCTION

Liver cancer is the third leading cause of cancer-related death globally, accounting for about 906 000 new cases and 830 000 deaths worldwide [1]. The incidence and mortality rates keep increasing in the United States [2]. According to the American Cancer Society, the five-year survival rate for liver cancer is only 20%, the second lowest among all cancers [2]. Due to the limitations of current diagnostics, focal liver lesions (FLLs) are often found incidentally and many patients with malignant FLLs are thus diagnosed in an advanced stage [3], [4]. Early differentiation between benign and malignant FLLs is thus of uttermost importance so that appropriate treatment may be initiated. Benign lesions include focal nodular hyperplasia, cysts, adenomas, and hemangiomas, as well as a wide variety of regenerative and dysplastic nodules seen in patients with cirrhosis, while malignant lesions include hepatocellular carcinoma (HCC), accounting for 75%–85% of all liver cancers, cholangiocarcinoma, biliary cystadenocarcinoma, and hepatic metastasis [5], [6].

Imaging plays an important role in the diagnosis and management of liver cancer [7]–[11]. As standardized by the American College of Radiology Liver Imaging Reporting and Data System (ACR LI-RADS) for patients at risk of HCC [8], surveillance is performed by ultrasound (US), followed by classification that is performed by computed tomography (CT), magnetic resonance imaging (MRI), or contrast-enhanced ultrasound (CEUS).

Differently from CT and MRI, contrast agents used for CEUS are purely intravascular and allow for real-time assessment of the vascular enhancement patterns without the use of ionizing radiation at higher spatial and temporal resolution [9]–[11]. Moreover, since they are not nephrotoxic, they are safe to use in patients with renal insufficiency [9], [10].

Current assessment of FLLs with CEUS is performed by purely qualitative evaluation of the vascular enhancement patterns, with emphasis on presence, type, and degree of arterial phase enhancement of the FLL, as well as presence, timing, and degree of contrast wash-out [8], [11], [12].

Besides requiring expertise in both acquisition and interpretation [8], [13], current qualitative evaluation of CEUS images acquired at the different phases is a labor-intensive and time-consuming task, which could be affected by inter-reader variability [14], [15]; moreover, it does not fully exploit the rich spatiotemporal information present in the CEUS images. Over the years, several methods have been developed to extract quantitative information from CEUS, ranging from analysis of time-intensity curves (TICs) to full spatiotemporal assessment of the contrast agent transport [16], [17]; however, in the liver, motion artifacts due to probe and respiratory motion make quantitative analysis of CEUS challenging [18], [19].

A number of methods have been proposed to quantitatively analyze CEUS images for computer-aided FLLs characterization [15], [20]–[28]. Initial attempts mainly focused on assessing the temporal characteristics US contrast enhancement by analysis of TICs [15], [20], [22], [23]. In a parametric imaging approach, dynamic vascular patterns were mapped into a color-coded image by classifying each pixel TIC in the lesion into four distinct vascular signatures, based on comparison with the average TIC in an adjacent region-of-interest (ROI), representative of the liver parenchyma [20]. Compared to visual inspection of the full CEUS exam, interpretation of the obtained parametric images by radiologists showed superior diagnostic performance and better interobserver agreement [15]. Machine learning methods were also proposed for automatic TIC interpretation and FLLs characterization, achieved either by first extracting features from analysis of TICs in the lesion core, lesion periphery, and parenchyma, and then feeding these features to an artificial neural network (ANN) [22], or by an end-to-end approach combining automatic TIC extraction from the arterial and portal phases by factor analysis of dynamic structures with a deep-belief network for classification of the lesion into benign or malignant [23].

Focusing on spatial characteristics at different temporal phases, Liang *et al.* [26] proposed a fully automated method, which first trains several local classifiers to find discriminative ROIs in the arterial, portal, and late phases, from which spatial features are then extracted by texture and local-phase analysis and used for lesion classification. In a study by Huang *et al.* [27], spatial semantics extracted at multiple frames by analysis of local binary patterns also proved useful for differentiation of atypical HCC.

Spatial and temporal characteristics have also been combined by adding to the temporal features, obtained by TIC analysis, a set of spatial features, obtained by analysis of the image intensity spatial patterns at different temporal phases of the CEUS cines [21], [24], [25] and the B-mode image prior to contrast injection [24]. The obtained features were then concatenated and fed to machine learning models such as ANNs [24] or support vector machines (SVMs) [21], [25] for different classification tasks.

Recently, a method based on convolutional neural networks was also proposed to achieve end-to-end lesion classification, avoiding the need for hand-engineered feature extraction [28]. The method, however, still requires the manual selection of the lesion ROI by an expert radiologist, based on which 50 ROIs from each vascular phase (arterial, portal, and late) are then manually selected.

One major limitation of previous studies is the lack of analysis and interpretation of the importance of each feature in producing the output prediction, which could offer useful clinical insights and provide a better understanding of the decision process, possibly allowing for debugging and improvements at all steps of the acquisition and processing chain. Moreover, most of the methods require a motion compensation step [15], [20]–[22], [25] and the definition of a parenchyma ROI prior to feature extraction [15], [20], [22], [24], [25]. Motion compensation is notoriously challenging in liver CEUS, requiring careful fine-tuning and appropriate validation [19]; for currently standard 2-D CEUS acquisitions, the presence of out-of-plane motion makes motion compensation even more challenging and often leads to discarding a large number of frames [18], [19]. Delineation of an appropriate ROI to represent the lesion parenchyma, either manually or automatically, is also a critical step, as the ROI should ideally be at the same imaging depth of the lesion ROI to avoid depth-dependent differences in image intensity and should also avoid areas with large vessels; these conditions are often difficult to meet for large and highly vascularized lesions. Some methods additionally require long acquisition times up to 30 min [21], [24], [25] to observe the postvascular phase and the injection of a second bolus followed by a high-pressure US pulse to sample the replenishment curve by inflowing microbubbles [24]. More complex models can achieve end-to-end classification, avoiding the need for all preprocessing and feature extraction steps, but often require large patient dataset due to the large number of trainable model parameters [26], [28] and they are generally more difficult to interpret [23], [26], [28].

In this work, we propose an interpretable machine learning approach to differentiate benign and malignant FLLs on CEUS, requiring minimal manual input and avoiding the need for motion compensation. Our approach is based on the extraction of features that are relatively robust to motion. Similar to previous work, this is achieved by spatial and spatiotemporal analysis of the CEUS loops; however, our method differs from existing work in a number of aspects. First, we intentionally avoid the use of model fitting for TIC analysis, as the presence of motion and the overlap between the arterial and portal phase generally make model fitting unreliable. Second, we propose for the first time in the liver a set of CEUS-based spatiotemporal features to capture simultaneously TIC characteristics in time and space [29], [30]. Third, we focus on short CEUS acquisitions of about 60 s, capturing mainly the arterial phase and part of the portal phase, thus avoiding the need for long examination times and a double contrast injection. Fourth, we implement an automatic method for selection of the most suitable frames for spatial analysis at three different phases of the short CEUS cine based on their spatial correlation with a reference frame, making them more robust to motion. Finally, we perform the analysis and interpretation of the feature relative importance for the output prediction, possibly providing useful insights for clinical decision-making and the further optimization of computer-aided diagnostic methods.

## II. METHODS

### A. Patient and Data Acquisition

This HIPAA-compliant study was approved by the Institutional Review Board of all participating institutions. Informed consent was obtained from each patient prior to data collection. All data were deidentified and pseudonymized prior to analysis.

**Fig. 1.**
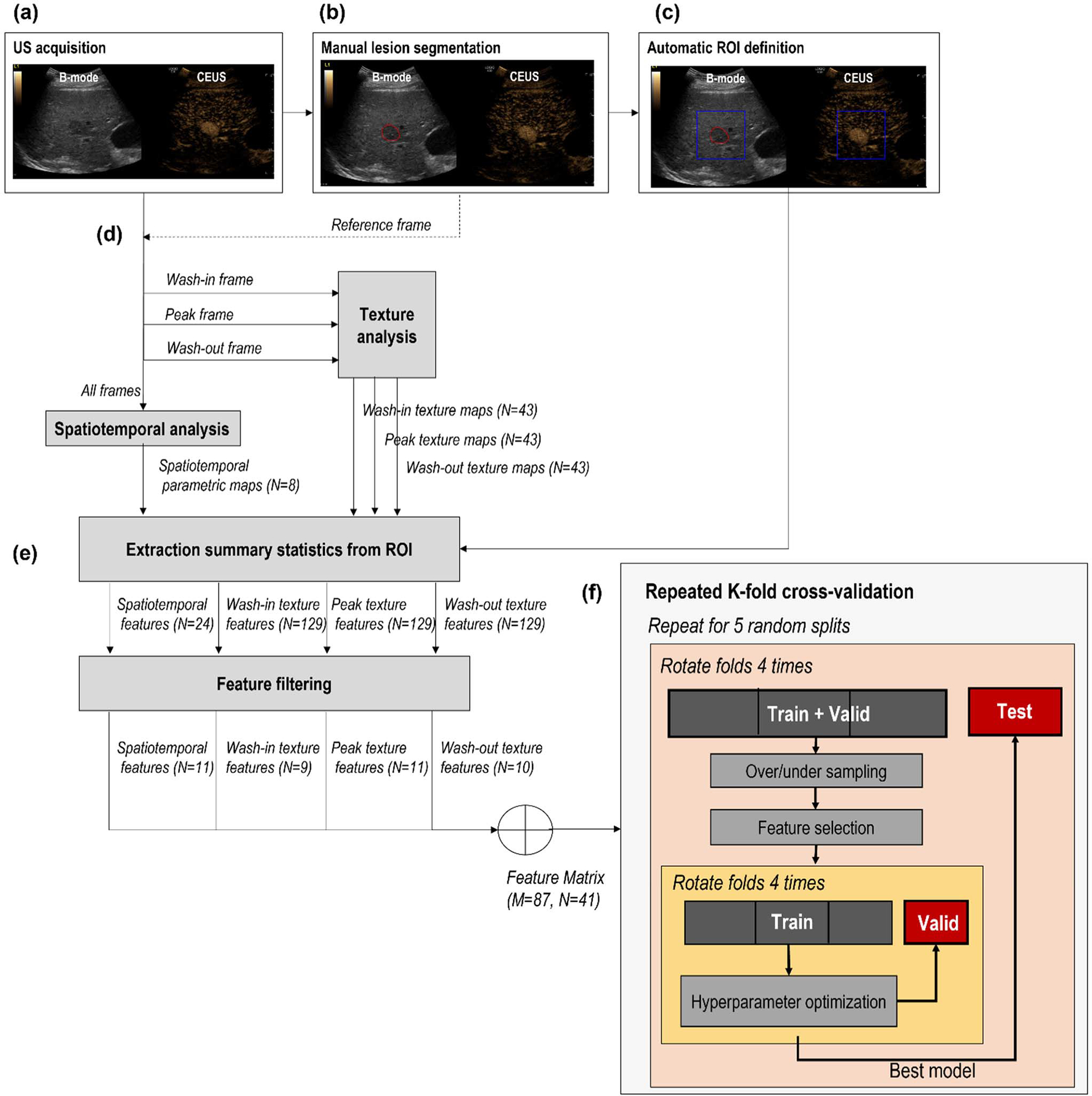
Flowchart describing the processing and machine learning pipelines. (a) Side-by-side view of B-mode and CEUS. (b) Manual segmentation of the lesion on the B-mode image. (c) Automatic definition of the ROI based on the location of the manually drawn lesion. (d) Extraction of spatiotemporal features (using all frames) and texture features at wash-in, peak, and wash-out frames. (e) Extraction of summary statistics from ROI and feature filtering for dimensionality reduction; *N* represents the number of selected features at each step, while *M* represents the number of samples. (f) Repeated nested *k*-fold cross-validation procedure for hyperparameter tuning (inner loop, yellow) and performance evaluation (outer loop, orange).

The study included 72 patients at risk of HCC who were examined for evaluation of FLLs at the Thomas Jefferson University Hospital (Philadelphia, PA, USA) or the Stanford University Medical Center (Stanford, CA, USA). CEUS was performed by injection of a bolus of Lumason, according to the ACR CEUS LI-RADS Working Group [31]. Recordings of approximately 60 s were obtained to visualize the contrast wash-in, peak enhancement, and beginning of wash-out. Each recording presented a side-by-side view of the B-mode and the contrast-specific acquisition [see Fig. 1(a)]. When multiple FLLs were present, up to two of the most visible lesions were investigated. Each lesion was examined independently and considered an independent sample, as different lesion types can be present in the same patient. All CEUS investigations were assessed by board-certified body/abdominal imaging radiologists and the final diagnosis was obtained by further evaluation with CT, MRI, and/or histopathology. A total of 87 lesions were included, of which 13 were benign and 74 malignant. Malignant lesions included 71 HCC and 3 intrahepatic cholangiocarcinoma (ICC). Details on the US acquisition settings and the breakdown for different lesion types of CEUS LI-RADS, liver disease etiology, and cirrhosis are provided in Tables S1–S4, respectively (see the Supplementary Material).

### B. Data Processing and Feature Extraction

Fig. 1 schematically shows the data processing and feature extraction pipeline. For each lesion, the US examination consisted of a cine loop with a side-by-side view of the B-mode acquisition next to the CEUS acquisition. The lesion was first manually delineated on the B-mode window, and a square ROI was then automatically obtained around the lesion. In parallel, CEUS loops were first linearized based on the known dynamic range [32] and then quantitatively analyzed by spatiotemporal and texture analysis to obtain pixel-based parametric maps. Using a radiomic approach, the parameter values in the ROI were condensed by extracting summary statistics. These were finally used as input to the machine learning models. The processing pipeline is further detailed hereafter. Data processing and feature extraction was performed on MATLAB (The MathWorks Inc, Natick, MA, USA) version 9.8.0.1323502 (2020a).

#### 1) Lesion Delineation and ROI Definition:

For each lesion, a certified radiologist indicated the location of the lesion on one frame of the US cine, where the lesion was well visualized, as shown, for example, in Fig. 1(a). Based on this indication, each lesion was segmented manually on the B-mode side of the CEUS loop by using ITK-SNAP [33] [Fig. 1(b)]. The manual segmentation was performed in 1–3 frames where the lesions were clearly visible to increase robustness toward motion artifacts. In fact, motion is unavoidably present in liver CEUS cines due to US probe displacement, respiration, and other physiologic movements [19]. Based on the manually segmented frames, a square ROI was automatically selected, centered around the center of mass of manual delineation(s), and included also part of the structures surrounding the lesion [Fig. 1(c)]. The ROI size ranged from 200 × 200 to 278 × 278 pixels, that is, 2.74 × 2.74 cm^2^ to 8.24 × 8.24 cm^2^, depending on the actual lesion size in centimeters.

#### 2) Spatiotemporal Analysis:

CEUS is an established modality for assessment of microvascular perfusion [16], [17]. From the TIC analysis, several semiquantitative and quantitative parameters can be extracted that are related to blood flow and volume and the microvascular architecture [16]. Generally, it is advisable to fit the obtained TICs to suitable indicator dilution models prior to parameter extraction, as it increases robustness to noise, provides more accurate parameter estimates, and allows for the estimation of parameters that are more directly related to the underlying physiology [16]. However, in the liver, the arterial phase, which starts at 10–20 s and ends at 30–45-s postcontrast injection, partially overlaps with the portal phase, which starts at 30–45 s [11], hiding the contrast wash-out in the arterial phase. Thus, in this work, we intentionally avoided the use of TIC model fitting, as the overlap between the arterial and portal phases at the beginning of contrast wash-out, together with the unavoidable presence of motion, makes model fitting unreliable. Instead, focusing on the arterial phase, we directly extracted semiquantitative parameters from the TIC. After preprocessing by a 5-s moving average filter, the following parameters were extracted: peak intensity, peak time, appearance time, wash-in time, and wash-in rate [34]. These are further defined in Table I.

To go beyond temporal TIC analysis, we also performed a spatiotemporal similarity analysis by comparing each pixel TIC with neighboring TICs in a ring kernel [29], [30], with an inner radius of 1 mm and an outer radius of 2.5 mm.

These dimensions were chosen based on the known tumor size limit of 1 mm^3^ in volume (1.25 mm in diameter, assuming a spherical volume), after which the formation of a vascular network is required to permit further tumor growth, a concept known as angiogenesis switch [30], [35]. Linear similarity was quantified in the frequency domain by the spectral coherence and in the time domain by the linear correlation [30]. As described in [30], prior to similarity analysis, anisotropic spatial filtering for speckle regularization is performed, and a time window is selected, starting from the bolus appearance time. In this work, however, we reduced the time window to 20 s to focus on the arterial phase only, avoiding confounding effects from the overlapping portal phase. In addition, the mutual information between neighboring TICs was estimated as a measure of nonlinear similarity, as described in [30], and again reducing the time window to 20 s.

#### 3) Texture Analysis:

**Fig. 2.**
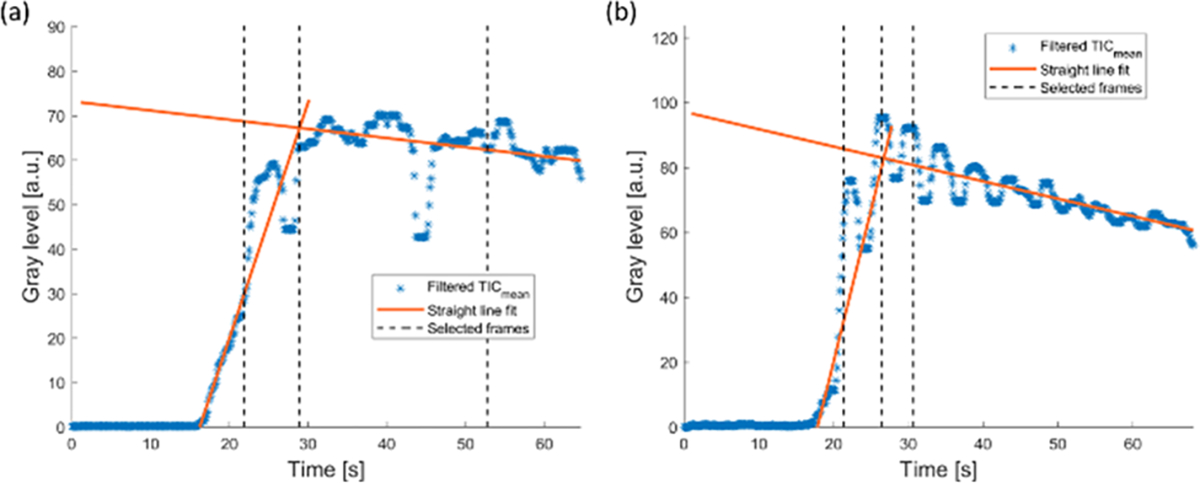
Two examples of average TIC (TIC_mean_) obtained from the lesion ROI (blue stars), together with the straight-line fit in the wash-in and wash-out (orange solid lines). The times at which the wash-in, peak, and wash-out frames were selected are indicated by dashed vertical lines.

Spatial information at different vascular phases was extracted by applying texture analysis at three different frames of the CEUS loop, selected at fiducial time points during contrast wash-in, peak intensity, and contrast wash-out. To make the search for these time points more robust toward motion artifacts, first, the correlation between the reference frame, i.e., the frame which the radiologist used to indicate the lesion, and all other frames was calculated on the B-mode images. Only frames with a correlation higher than the empirically chosen threshold of 0.8 were considered valid, thus filtering out frames with large motion artifacts. The B-mode was only used for the selection of the reference frame and valid frames, while the rest of the analysis was performed on the CEUS data. A mean TIC (TIC_mean_) was calculated on the CEUS loop by averaging over all pixels in the manually segmented lesion at the reference frame. A straight line was fit to the TIC_mean_ wash-in, in the interval defined by the time points at which the TIC_mean_ intensity is between 5% and 50% of the intensity at peak, using only valid frames. Similarly, a straight line was fit to the wash-out in the second half of the time window defined from the appearance time to the end of the recording (Fig. 2). The intersection between these two straight lines was then used to find an initial guess for the peak time. The peak frame was found as the frame at which TIC_mean_ was maximum, in a window including 20 samples before and 50 samples after the initial peak guess, including only valid frames. The wash-in frame was defined as the frame with the highest correlation with the reference frame, in a window starting five samples after the appearance time and ending five samples before the peak time. Finally, the wash-out frame was defined as the frame with the highest correlation with the reference frame, in a window starting ten samples after the peak time until the end of recording.

**Fig. 3.**
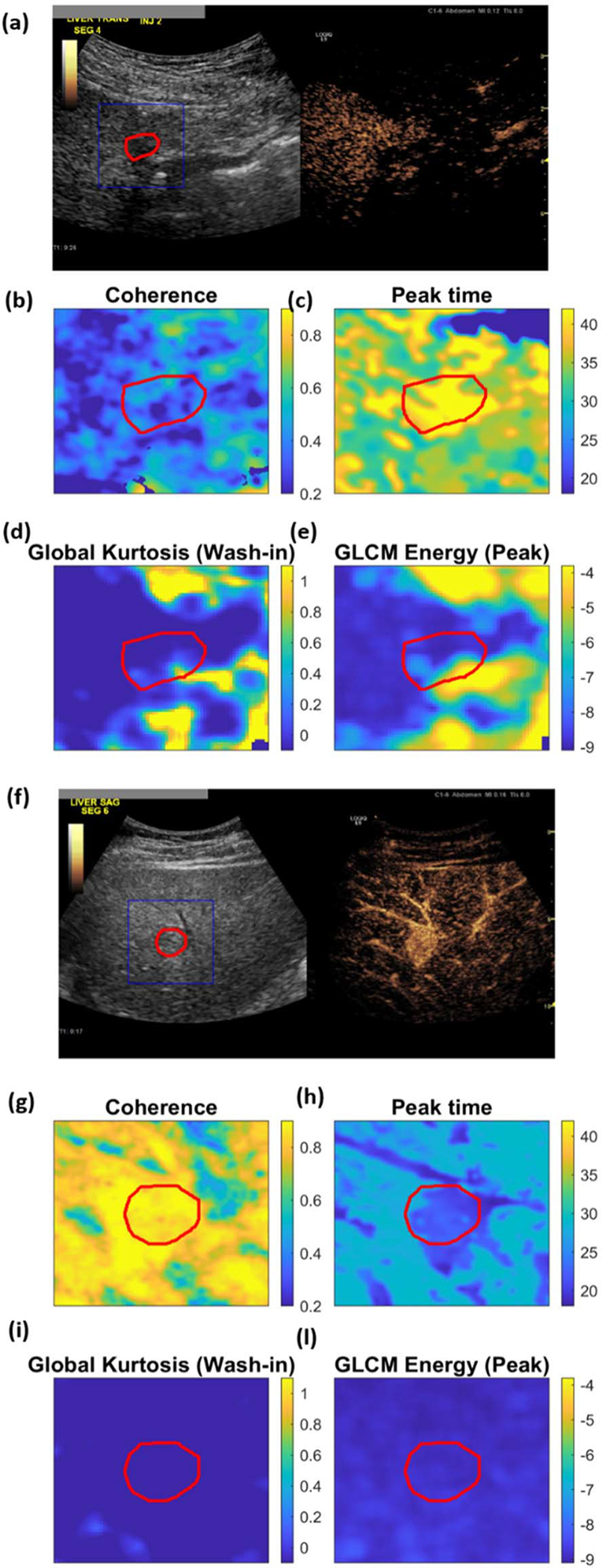
(a)–(e) Examples of parametric maps obtained for one benign and (f)–(l) one malignant lesion: (a) and (f) side-by-side view of B-mode and CEUS at the reference frame, with manually delineated lesion and the analysis ROI highlighted in blue and red, respectively; (b) and (g) spatiotemporal feature “Coherence,” (c) and (h) spatiotemporal feature “peak time”; (d) and (i) texture feature “Global Kurtosis” at wash-in; (e) and (l) texture feature “GCLM Energy” at peak (visualized in logarithm scale).

Once the wash-in, peak, and wash-out frames were defined based on the fiducial time points, texture analysis was performed separately for each of these frames by using the texture feature extraction module of the radiomics MATLAB toolbox implemented by Vallières *et al.* [36], [37]. Typically, this analysis requires the definition of an ROI, from which a single value for each texture feature is calculated. In order to retain the local characteristics of the features, possibly highlighting structures with different textures in the image, here, we performed the analysis defining the ROI as a moving window. In this way, parametric maps, showing the texture feature values at each imaging pixel, could be obtained (see Fig. 3). The window was chosen of size 21 × 21 pixels and was moved with a stride of 3 pixels. These settings were optimized empirically to balance between excessively noisy or smooth feature maps, preserving at best structures with different textures. Prior to feature extraction, the intensity range in the window was quantized to 64 gray levels, as described in [36]. The 43 texture features summarized in Table II were extracted for each of the three selected frames (wash-in, peak, and wash-out). Global features are calculated from the histogram of the intensity values in the ROI, while the rest of the features are calculated from matrices estimated by calculating the second (GLCM) and higher order spatial statistical properties (GLRLM, GLSZM, and NGTDM) of an image. These features are calculated on the CEUS frames at wash-in, peak enhancement, and wash-out. In the results, these are indicated by prefixes “WiIm,” “PkIm,” and “WoIm”, respectively. A detailed description of all texture features can be found in [37].

#### 4) Summary Statistics:

After spatiotemporal and texture analysis, a total of 137 parametric maps were obtained, including spatiotemporal features (*N* = 8), wash-in texture features (*N* = 43), peak texture features (*N* = 43), and wash-out texture features (*N* = 43) [Fig. 1(d)]. To summarize the information in the selected ROIs, summary statistics of the parameter values over the ROI were extracted for each feature [Fig. 1(e)].

Since the features were generally not normally distributed, we calculated the median, interquartile range, and skewness. In the results, these are indicated by the suffixes “median,” “iqr,” and “skew.” Each summary statistic was treated as a separate feature, thus obtaining 411 features for each lesion.

### C. Machine Learning

Given the large number of features, feature filtering was performed prior to optimization and training of the machine learning models, in order to reduce the dimensionality of the problem [Fig. 1(e)]. First, correlation analysis was performed to remove features that are highly correlated, with the goal of reducing information redundancy. For each pair of features with correlation higher than 0.9, the feature with the highest correlation with the label (malignant/benign) was kept, while the other was discarded. Then, univariate feature selection was performed to pick the best *N* features, based on the mutual information with the label. This operation was performed separately for each set of features (spatiotemporal, wash-in texture, wash-out texture, and peak texture features), choosing *N* adaptively for each feature set by performing principal component analysis and calculating the number of components necessary to explain 95% of the variance. These feature-filtering steps enabled reducing the number of features from 411 to 41.

The 41 filtered features were then used to train different machine learning models. Given the small dataset, optimization, feature selection, and performance evaluation were carried out by a repeated nested *k*-fold cross validation, which has shown to produce conservative estimates of the model performance [38]. As shown schematically in Fig. 1(f), the dataset was first split into four folds, of which three were used as training + validation set and one as test set; the training + validation set was further divided into three folds used for tuning the hyperparameters (training) and one fold used to choose the best model (validation); finally, the performance was tested on the test set. For both the inner and outer cross-validation procedures, the folds were rotated four times so that each fold was once in the validation/test set. The whole procedure was then repeated five times for five different random splits of the lesions in the outer four folds, thus obtaining a total of 20 evaluations of the model performance. To cope with the unbalanced dataset, a synthetic minority oversampling technique (SMOTE) [39] was used to oversample the benign cases (minority class) to a ratio of 0.5 of the number of malignant cases (majority class). The malignant cases were then randomly undersampled to a ratio of 0.7 of the original size. This operation was only applied on the training set and never on the test set since the performance evaluation should not be calculated on artificial data to avoid overoptimistic results [40]. Backward sequential feature selection was then performed to further reduce the features to an optimal number, which was optimized separately for each classifier. The hyperparameters were tuned in each fold on the training set by further splitting this set in four folds and performing a cross-validated grid search [41]. The best hyperparameters were chosen as the most occurring ones over all folds. The procedure in Fig. 1(f) was then repeated by fixing the hyperparameters at their optimal values and calculating the model performance over the 20 total repetitions (four folds rotations by five random splits) of the cross-validation procedure. All the steps in the machine learning pipeline [Fig. 1(e) and (f)] were carried out on Python (version 3.8.8), using the Scikit-learn library [41].

#### 1) Machine Learning Models:

Several machine learning models were trained and optimized, including logistic regression (LR), support vector machine (SVM), random forest (RF), and *k*-nearest neighbor (kNN) [42]. An overview of the hyperparameters that were optimized for each classifier is provided in Table III. Since the dataset is unbalanced, the optimization was performed to maximize the balanced accuracy (bACC), which is given by

(1) $$\text{bACC}=\frac{1}{2}\left(\frac{\text{TP}}{\text{TP}+\text{FN}}+\frac{\text{TN}}{\text{TN}+\text{FP}}\right)$$

where TP, TN, FP, and FN are the number of true positives, true negatives, false positives, and false negatives, respectively. Here, a malignant lesion is regarded as positive, while a benign lesion is regarded as negative. The bACC can also be interpreted as the average between sensitivity (SENS) and specificity (SPEC) [43].

The optimized models were then used to implement a voting classifier, which combines the predictions of different classifiers by majority voting (hard voting) or by calculating the weighted average of the output probabilities (soft voting). In this work, we implemented a soft voting classifier (sVC) by combining all the models in Table III, using the optimized hyperparameters.

#### 2) Feature Selection and Interpretation:

In each fold, the optimal feature subset was found by backward sequential feature selection. This procedure removes features sequentially by keeping at each step the subset of *M*-1 features that gives the highest classification accuracy, with *M* the number of features at the previous step. The procedure stops when the desired number of features is obtained. To select the optimal number of features, each classifier was trained repeatedly by changing each time the desired number of features as input to the feature selection procedure. A number of features ranging from 12 to 41 (full set) were investigated. In addition, feature relevance was assessed by calculating the frequency of being chosen and the permutation feature importance (PFI) for each feature over the 20 repetitions of the cross-validation procedure. For a feature, the PFI is calculated as the decrease in model performance when the values of that feature are randomly shuffled, losing any relationship with the output class. This technique has the advantage to be model-agnostic and thus facilitates the comparison between different models.

#### 3) Model Evaluation:

The performance of the classifiers for distinguishing between benign and malignant FLLs were compared by calculating the mean and standard deviation of the accuracy (ACC), bACC, SENS, and SPEC, and area under the receiving operator characteristic curve (AUC_ROC_) averaged over the five random splits of the four-fold cross-validation procedure, for a total of 20 performance evaluations. The statistical significance of the difference in the obtained performance was calculated by running a *k*-fold cross-validated *t*-test as described in [44]. However, since our *k*-fold procedure was repeated five times for five different random splits of the data in fourfold, we additionally applied a correction in the variance estimate used to calculate the *t*-statistic, as suggested by Nadeau and Bengio [45]. Let $p^{\left(i\right)}=p_{A}^{\left(i\right)}-p_{B}^{\left(i\right)}$ be the difference in a given performance metric at repetition *i* between models *A* and *B*. The corrected *t*-statistic of *p* is calculated as

(2) $$t=\frac{\bar{p}}{\sqrt{{\hat{\sigma }}_{\text{corr}}^{2}}}=\frac{\bar{p}}{\sqrt{{\hat{\sigma }}^{2}\left(\frac{1}{n}+\frac{n_{1}}{n_{2}}\right)}}$$

where $\bar{p}=(1/n)\sum_{i=1}^{n}p^{\left(i\right)}$, *n* is the total number of repetitions, *n*_1_ is the number of samples in the training set, *n*_2_ is the number of samples in the test set, and ${\hat{\sigma }}^{2}$ is the estimated variance, calculated as the sample variance *S*_*n*_ divided by *n* as

(3) $${\hat{\sigma }}^{2}=\frac{S_{n}}{n}=\frac{\sum_{i=1}^{n}{\left(p^{\left(i\right)}-\bar{p}\right)}^{2}}{n(n-1)}.$$

Under the null hypothesis that the performance of models *A* and *B* are not different, the *t*-statistic has a *t*-distribution with *n* − 1 degrees of freedom. The null hypothesis can be rejected with level of confidence *α* = 0.05 if |*t*| *> t*_*n*−1,0.975_. Here, the chosen metric to compare the performance is the bACC.

## III. RESULTS

### A. Feature Extraction and Preprocessing

Fig. 2 shows one example of the procedure used to extract the wash-in, peak, and wash-out frames. Two straight lines (orange lines) are fit to the wash-in and the wash-out to find a guess for the peak frame. From this initial guess, the three frames at wash-in, peak, and wash-out with the highest correlation with the reference frame are then found and used for feature extraction (black dashed lines).

Fig. 3(a)–(e) and (f)–(l) shows examples of the extracted parametric maps in one benign and one malignant lesion, respectively, including the spatiotemporal features “coherence” [Fig. 3(b) and (g)], = and “peak time” [Fig. 3(c) and (h)], the texture feature “Global Kurtosis” at wash-in [Fig. 3(d) and (i)], and the texture feature “GCLM Energy” at peak [Fig. 3(e) and (l)], with the lesion segmentation and analysis ROI highlighted in red and blue, respectively. The histograms of the features shown in Fig. 3 can be found in the Supplementary Material (Fig. S1).

When comparing the texture feature maps with the grayscale CEUS image of the reference frame [Fig. 3(a) and (f)], it can be seen that most of the structures in the images are preserved. Feature values for the benign and malignant lesion present differences in both the lesion and the surrounding parenchyma. Both cases presented with alcoholic liver disease, but cirrhosis was present only in the benign case. Cirrhosis might thus contribute to the differences observed in the parenchyma.

### B. Machine Learning Models

The performance in distinguishing between malignant and benign FLLs is shown in Table IV in terms of ACC, bACC, SENS, SPEC, and AUC_ROC_. The results are reported as the average over the 20 repetitions of the cross-validation procedure, with standard deviation given in parenthesis. Although the sVC classifier gave higher performance for all metrics, with lower standard deviation, the increase in performance was not significantly different, as tested by the corrected *k*-fold cross-validated *t*-test. To highlight the problem of the dataset imbalance, the performance is also compared with a “Naïve” classifier, obtained by predicting every lesion as malignant. This would give a comparable accuracy and significantly higher sensitivity, but the bACC will be very low and the specificity would obviously be zero.

### C. Feature Selection and Interpretation

**Fig. 4.**
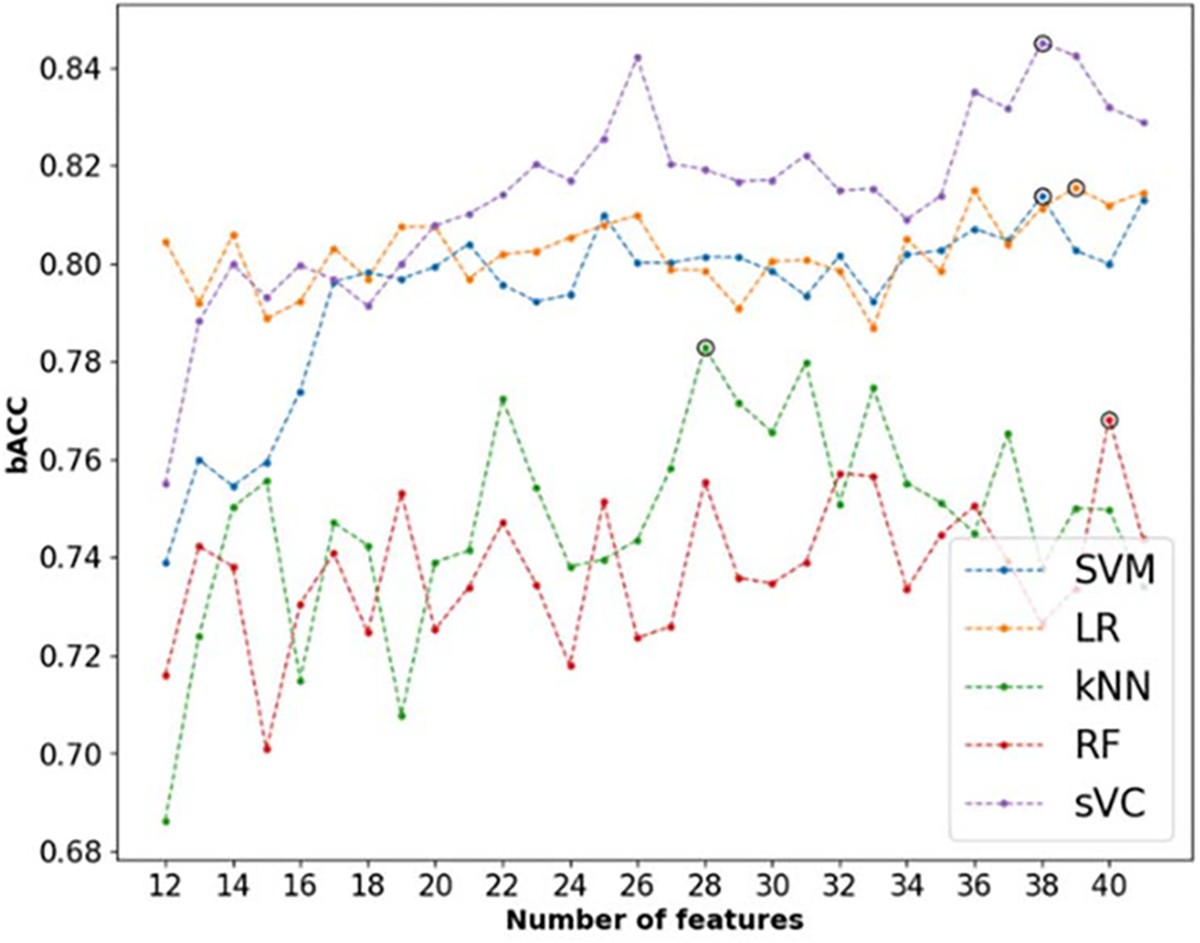
Analysis of the number of features required to optimize the bACC for each model. The optimal number for each classifier is highlighted with a black circle.

The number of selected features was optimized by running the cross-validation procedure for a different number of features and picking the number of features that gave the highest bACC. This is shown in Fig. 4 for each classifier, with the optimal number of features highlighted with a black circle. While LR, SVM, and sVC are relatively stable for a number of features larger than 18, with small improvements with a larger number of features, larger variability is observed for the kNN and RF. For the latter, this can be explained by the nature of the RF algorithm, which trains at each iteration a number of different trees with randomly selected features. Because of this built-in feature selection mechanism, tree-based algorithms often do not benefit from feature selection [46].

**Fig. 5.**
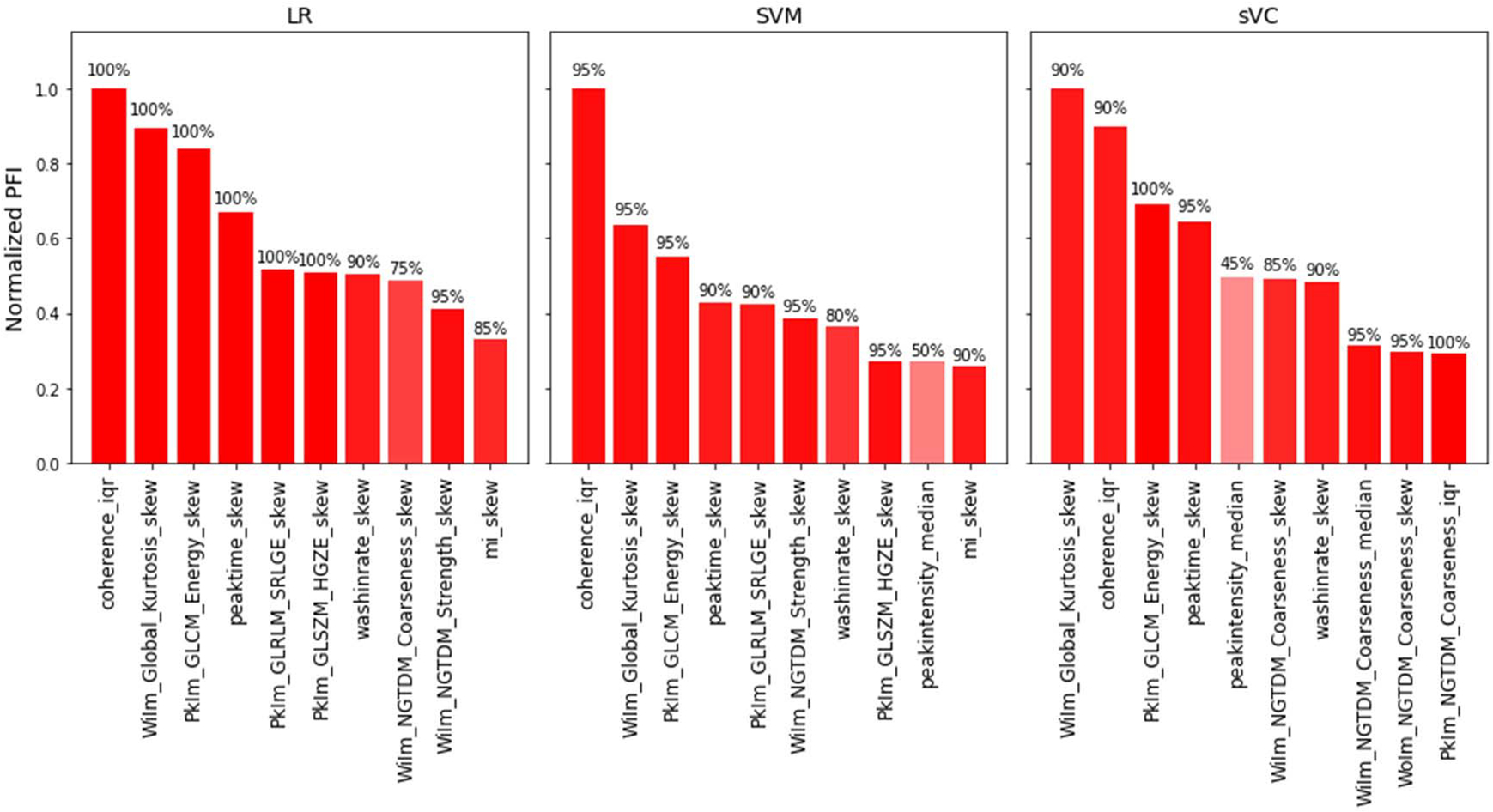
Normalized PFI of the top 10 features for the LR, SVM, and sVC classifiers. The percentage of times that a feature was chosen is given by the text on top of each bar and additionally indicated by the shading of the bars. For the texture features, the prefixes “WiIm,” “PkIm,” and “WoIm” indicate features extracted at wash-in, peak intensity, and wash-out, respectively. For all features, the suffixes “median,” “iqr,” and “skew” indicate the median, interquartile range, and skewness, respectively, extracted over the ROI.

To understand the contribution of each feature to the output prediction, we calculated the PFI of the selected features at each fold. In Fig. 5, the average normalized PFI of the top 10 features over the 20 *k*-fold repetitions is shown for the three best performing classifiers, namely, LR, SVM, and sVC. In addition, the shading and text in each bar indicate the percentage of times that each feature was selected. For all classifiers, the most relevant features include a combination of spatiotemporal and texture features extracted at wash-in and peak. For almost all the top features, the skewness and interquartile range were more relevant than the median value in the ROI.

A 3-D scatter plot of the three top features (Coherence_iqr, WiIm_Global_Kurtosis, and PkIm_GLCM_Energy) can be found in the Supplementary Material (Fig. S2). A cluster of malignant samples can already be observed, using only three features.

## IV. DISCUSSION

Our results show that combining spatiotemporal features and texture features extracted at fiducial time points by machine learning has the potential for computer-aided characterization of FLLs in patients at risk for HCC, with no need for motion compensation and requiring minimal manual input. Our strategy aims at extracting features that are relatively robust to motion. As the texture features are based on the analysis of spatial statistical properties at specific time points, these features are generally less affected by motion artifacts. However, to ensure that the investigated lesion is approximately in the same position in all the selected frames, we further proposed an automated procedure to find fiducial time points in each phase, at which the correlation with the reference frame was the highest.

For the spatiotemporal features, a set of features were extracted by the model-free temporal analysis of TICs, limited in the time window up to peak intensity, thus avoiding the overlap with the portal phase. Although TICs are affected by noise and motion artifacts, causing amplitude variations, the time-dependent TIC features, as well as features depending on the ratio between amplitude and time, have shown to be more robust to noise compared to features that are purely amplitude-based [47]. In line with these findings, our analysis of feature relevance shows that the peak time is more important for FLLs classification by our proposed method compared to other TIC features that are dependent on the ratio between amplitude and time (wash-in rate) or on amplitude alone (peak intensity). The rest of the spatiotemporal features were extracted by the similarity analysis of TICs in a ring kernel. By this procedure, each pixel TIC is compared with all the TICs in the kernel to calculate linear and nonlinear similarity measures. Since motion artifacts affect the TICs in a local neighborhood in a similar manner, we assume that the influence of motion on the extracted similarity measures is limited. Moreover, to focus on the arterial phase, the analysis window was limited to 20 s from the contrast appearance time, limiting the overlap with the portal phase. While the promise of spatiotemporal similarity analysis has been demonstrated in previous studies on CEUS imaging of prostate cancer [29], [30], [48]–[50], this is the first time that this approach is translated to another human organ. Similarity analysis permits the quantification of local parameters reflecting the contrast agent dispersion kinetics, which have been related to the tortuosity of tumor microvasculature [30], [50]. In this study, linear similarity quantified in the frequency domain by the spectral coherence resulted to be one of the most relevant features for FLLs characterization, confirming the feasibility and promise of this approach for cancer diagnostics by CEUS. However, further investigation and optimization should be performed to ensure appropriate and efficient translation of spatiotemporal similarity for analysis of liver CEUS.

Besides achieving similar performance with different classifiers, model-agnostic feature importance analysis by the quantification of the PFI further shows that the most important features are stable across the best performing classifiers. Notably, for all classifiers, a mix of spatiotemporal features and texture features at wash-in, peak, and wash-out are always among the most important, reinforcing the evidence that the analysis of different phases of the CEUS cine contributes to improved FLLs diagnosis. However, only one wash-out texture feature was among the top 10 for the sVC, and none for the rest of the best performing classifiers. This might be influenced by the frame selection procedure for the texture analysis. In fact, after peak enhancement, the correlation with the reference frame is often lost due to the overlap with the portal phase, and the occurrence of large motion artifacts due to patients taking deep breaths after a period of shallow breathing or breath hold. As a result, in order to ensure high correlation with the reference frame, the frame selection procedure often results in selecting a frame that is very close to the peak frame, which might lead to high correlation between peak and wash-out texture features, possibly making the wash-out features redundant [see Fig. 2(b)]. This could be improved by further optimization of the frame selection procedure, including, e.g., adaptive tuning of the correlation threshold. Moreover, in this preliminary study, we focused on short cines of about 1 min, visualizing only part of the arterial wash-out, which often overlaps with the beginning of the portal phase. As the contrast agent wash-out in the portal and late phase has shown to aid diagnosis [12], [51], [52], investigation of additional features extracted at later phases should be performed in the future. Repeated, short acquisitions (~10 s) of the wash-out at different time points, up to about 6 min, are in fact available for this dataset and could be exploited in the future. For instance, texture feature could be extracted at the different sampled phases of the wash-out; moreover, the degree of wash-out could be assessed by averaging the intensity in the lesion in each acquisition and fitting an exponential decay.

Among the most important texture features were the wash-in Global kurtosis and the peak GLCM energy. Global features are calculated from the histogram of gray levels and thus look only at the distributions of gray levels, without reflecting spatial structures in the image. The kurtosis is the fourth statistical moment of a probability distribution and can be interpreted as a measure of the relative weight of the tails, taking the normal distribution as a reference. Large kurtosis indicates that tail values are more extreme compared to a normal distribution. In our context, large wash-in global kurtosis may indicate that a small number of very large (high enhancement) and/or very small (low enhancement) gray values are present in the investigated ROI during the arterial phase. This can result from the presence of both localized strongly-enhancing and nonenhancing regions, as well as inhomogeneous enhancing regions in the arterial phase, which are known hallmarks of malignant FLLs [11]. The GLCM texture is a square matrix of dimensions equal to the number of gray levels in the ROI in which it is calculated. Each element (*i*, *j*) of the GLCM texture matrix is obtained by calculating the number of times that gray level *i* was neighbor with gray level *j* [53]. The GLCM energy is an intensity-invariant feature calculated by summing all the squared elements of the GLCM texture matrix. Intuitively, a region of interest with very homogeneous gray levels, or with gray levels that change very gradually in space, will result in a larger number of gray level co-occurrences in the GLCM matrix and thus in higher GLCM energy compared to a very heterogeneous image with rapidly changing gray levels, for which the number of gray levels co-occurrences will be smaller. As a practical example, the GLCM energy of an image consisting of random noise is zero. In our context, lower GLCM energy may occur in regions with inhomogeneous enhancement, such as rim-like and spoke-wheel enhancement patterns, or for regions including both hyperenhancing and nonenhancing areas. These enhancement features are observed in both malignant and benign lesions [11].

Previous research has shown that comparing the CEUS characteristics in the lesion with those in the parenchyma, either qualitatively or quantitatively, generally improves the diagnostic performance [15], [20], [22], [24], [25]. By our method, the selected ROI is centered around the lesion but generally includes also surrounding structures, such as liver parenchyma and large vessels. Interestingly, our results show that almost all most relevant features (Fig. 5) were given by the interquartile range or the skewness of the selected features, suggesting that the heterogeneity of the feature values in the selected ROI is more relevant than the median values. This suggests that the choice of a large ROI together with the extraction of high-order summary statistics may provide an indirect way of relating the lesion characteristics with those of the parenchyma. Moreover, it further supports the choice of the proposed moving-window strategy to extract local texture characteristics, enabling highlighting different structures in the ROI.

Comparing the obtained performance with similar methods for computer-aided diagnosis of FLLs, accuracies ranging from 85.8% to 91.8% have been reported [21]–[26], [28]. However, a fair comparison is difficult, as other methods might have different objectives [24], [26], [28], and they have been tested on a different population, sometimes using larger training datasets [22], [24]. In addition, they generally require extra dedicated procedures for compensation of in-plane motion, manual selection of out-of-plane frames, and selection of a parenchyma ROI [21]–[26], [28].

Current clinical assessment of CEUS by LI-RADS assigns each lesion to the following risk groups: benign (LR-1), probably benign (LR-2), intermediate risk for HCC (LR-3), probably HCC (LR-4), definite HCC (LR-5), and probably of definite malignant, not HCC-specific (LR-M). While LR-4 and LR-5 have shown to be highly predictive for HCC, the diagnosis of LR-3 and LR-4 lesions remains challenging. In fact, in our dataset, about 40% of LR-3 and 21% of LR-4 lesions were actually benign (see Table S2, Supplementary Material). Similar findings have been reported in a large multicenter study [54]. Given the different classification (binary versus multiclass), a direct comparison with the performance of our method is not possible. However, with a larger dataset, in the future, we could focus on LR-3 and L3–4 lesions and investigate whether the proposed method could aid the diagnosis of these challenging groups, possibly upgrading them to LR-5 or downgrading them to LR-1 or LR-2.

One of the main limitations of this study resides in the datasets, which was relatively small, including only 72 patients for a total of 87 FLLs, and more importantly very imbalanced, consisting of only 13 benign lesions compared to 74 malignant lesions. Of these, 71 resulted to be HCC and 3 only ICC; thus, any influence on the performance of the different CEUS enhancement patterns observed for different lesion types cannot be evaluated in this study. In addition, the ratio of malignant to benign cases does not reflect the actual prevalence of malignant cancers in the American population, which is estimated at 50%–57% [55], [56]. However, obtaining a larger number of full examinations of benign lesions, including CEUS, MRI/CT, and/or histopathological analysis, is inherently difficult, as benign lesions are typically kept at the surveillance stage and analyzed by conventional US only.

To cope with the small dataset, here, we performed nested *k*-fold cross validation, as this method has been shown to be robust toward overfitting while providing a good estimation of the diagnostic performance [38]. A number of folds equal to four were chosen so that three to four benign cases were included in the test set at each rotation of the *k*-fold validation procedure. For each classifier, the hyperparameters were optimized by further splitting the training + validation fold into three folds for training and one for validation, thereby avoiding overfitting on the test data on which the performance was evaluated [see Fig. 1(f)]. Moreover, we repeated the procedure for five different random splits of the lesions in the four folds so as to reduce the dependence of the estimated performance on the individual random split. To mitigate the influence of the imbalance in the number of benign and malignant cases, we further applied the SMOTE algorithm to up-sample the minority class, followed by random down-sampling of the majority class. In addition, the bACC was chosen as the metric to optimize all the classifiers. Besides coping with the imbalanced dataset, this choice is also motivated by the clinical objective of optimizing both sensitivity and specificity. In fact, while false positives may lead to unnecessary invasive treatment for patients with benign lesions, false negatives result in undetected cancer, hampering timely treatment.

Although the aim of this work was to provide a method for computer-aided diagnosis of FLLs, requiring minimal manual input with no need for motion compensation, more dedicated strategies to reduce the effects of noise and motion could be further investigated. Robotic arms with optical tracking are being proposed for US image guidance during radiotherapy and 4-D CEUS in the liver; such systems could be used to alleviate the effects of out-of-plane motion [57], [58]. In addition, advanced filtering strategies, such as singular value decomposition and robust principal component analysis, could be used to improve the accuracy of the estimated perfusion parameters from CEUS [59]. Motion compensation could in fact improve the quality of the extracted features, especially the ones derived from spatiotemporal analysis. Motion-compensated TIC could be fit to suitable indicator-dilution models, which may provide more accurate and reproducible quantitative parameters, less dependent on the operator and acquisition settings [16]. Since motion is similar among neighboring pixels, similarity analysis is more robust to motion; however, the presence of similar motion artifacts in neighboring pixels will also contribute to pushing all the similarity features to higher values and, therefore, it may reduce to some extent the sensitivity of the method. Motion compensation prior to feature extraction may further improve the discriminative power of similarity features.

## V. CONCLUSION

An interpretable radiomics approach was proposed for characterization of FLLs by CEUS by combining features extracted by spatiotemporal analysis and features extracted by texture analysis at different fiducial time points. The proposed method requires minimal manual input, with no need for motion compensation or the use of dedicated equipment (e.g., robotic arm). Promising results were obtained on a multicenter clinical validation, including 72 patients and 84 FLLs, achieving bACC of 84%. The analysis of feature importance shows that aspects related to perfusion (peak time and wash-in time), the microvascular architecture (spatiotemporal coherence), and the spatial characteristics of contrast enhancement at wash-in (global kurtosis) and peak (GLCM Energy) are particularly relevant to aid FLLs diagnosis.

## Supplementary Material

supp1-3161719

## Figures and Tables

**TABLE I:** Overview of Feature Extracted by Spatiotemporal Analysis

Analysis method	Parameter	Description
TIC temporal analysis	Peak intensity	Intensity of the peak in the TIC
TIC temporal analysis	Peak time	Time at which the peak intensity is reached
TIC temporal analysis	Appearance time	Time at which 10% of the peak intensity is reached
TIC temporal analysis	Wash-in time	Time between appearance time and peak time
TIC temporal analysis	Wash-in rate	Ratio between peak intensity and time-to-peak
Spatiotemporal similarity	Coherence	Spectral coherence between each pixel TIC and the neighboring TICs in a ring kernel
Spatiotemporal similarity	Correlation	Linear correlation between each pixel TIC and the neighboring TICs in a ring kernel
Spatiotemporal similarity	Mutual information	Mutual information between each pixel TIC and the neighboring TICs in a ring kernel

**TABLE II:** Overview of Feature Extracted by Texture Analysis

Texture type	Features	Number of features
Global	Variance, Skewness, Kurtosis	3
Gray-level co-occurrence matrix (GLCM)	Energy, Contrast, Correlation, Homogeneity, Variance, Sum Average, Entropy, Dissimilarity, Auto Correlation	9
Gray-level run-length matrix (GLRLM)	Short Run Emphasis (SRE), Long Run Emphasis (LRE), Gray-Level Non-uniformity (GLN), Run-Length Non-uniformity (RLN), Run Percentage (RP), Low Gray-Level Run Emphasis (LGRE), High Gray-Level Run Emphasis (HGRE), Short Run Low Gray-Level Emphasis (SRLGE), Short Run High Gray-Level Emphasis (SRHGE), Long Run Low Gray-Level Emphasis (LRLGE), Long Run High Gray-Level Emphasis (LRHGE), Gray-Level Variance, (GLV) Run-Length Variance (RLV)	13
Gray-level size zone matrix (GLSZM)	Small Zone Emphasis (SZE), Large Zone Emphasis (LZE), Gray-Level Non-uniformity (GLN), Zone-Size Non-uniformity (ZSN), Zone Percentage (ZP), Low Gray-Level Zone Emphasis (LGZE), High Gray-Level Zone Emphasis (HGZE), Small Zone Low Gray-Level Emphasis (SZLGE), Small Zone High Gray-Level Emphasis (SZHGE), Large Zone Low Gray-Level Emphasis (LZLGE), Large Zone High Gray-Level Emphasis (LZHGE), Gray-Level Variance (GLV), Zone-Size Variance (ZSV)	13
Neighborhood gray-tone difference matrix (NGTDM)	Coarseness, Contrast, Busyness, Complexity, Strength	5

**TABLE III:** Optimized Hyperparameters for Each Classifier

Classifier	Hyperparameter description	Optimized hyperparameters
LR	*C*: inverse of regularization strength	*C*=0.5
SVM	*Kernel*: type of decision function *C*: penalty of the error term *γ*: parameter of radial basis kernel function	*Kernel*: radial basis function *C*=50 *γ*= 0.001
RF	*N*_leaf_min_: minimum number of samples required to he at a lead node *N*_feat_max_: max number of features allowed to form each tree	*N*_leaf_min_ = 1 *N*_feat_max_ 0.2·*N*
kNN	*N*_n_: number of neighbors	*N*_n_= 5

LR = Logistic Regression; SVM = Support vector machine; RF = Random Forest; kNN = k Nearest Neighbour; *N*=total number of features; *N*= total number of features

**TABLE IV:** Classification Performance for All Classifiers, Given as Average Over the 20 Repetitions of the Train-Test Procedure. For Each Metric, the Standard Deviation Is Given in Parenthesis

	ACC	bACC	SENS	SPEC	AUC_ROC_
**LR**	0.75 (0.07)	0.82 (0.08)	0.73 (0.07)	0.90 (0.15)	0.82 (0.08)
**SVM**	0.75 (0.08)	0.81 (0.09)	0.73 (0.08)	0.90 (0.15)	0.81 (0.09)
**RF**	0.73 (0.09)	0.79 (0.09)	0.72 (0.10)	0.86 (0.17)	0.79 (0.09)
**kNN**	0.75 (0.07)	0.78 (0.13)	0.74 (0.08)	0.82 (0.27)	0.78 (0.13)
**sVC**	0.78 (0.07)	0.84 (0.08)	0.76 (0.08)	0.92 (0.15)	0.84 (0.08)
**Naive classifier**	0.74	0.42	0.85	0	-
