# Supplementary material for: Interpretable Machine Learning for Characterization of Focal Liver Lesions by Contrast-Enhanced Ultrasound
Source: IEEE Trans Ultrason Ferroelectr Freq Control. Author manuscript; Available in PMC 2022 Jun 12. (PMC9188683; doi:10.1109/TUFFC.2022.3161719)
Supplement: supp1-3161719 [file NIHMS1802122-supplement-supp1-3161719.docx]

**Supplementary tables and figures**

**Table S 1 Overview of the CEUS acquisition settings**

| Institution | US Scanner | Probe | Imaging frequency |
| --- | --- | --- | --- |
| TJHU (N=63) | GE Healthcare Logiq E9 | Curved linear | 3-4 MHz |
|  | GE Healthcare Logiq E10 | Curved linear | 3-4 MHz |
|  | Toshiba TUS-AI800 | Curved linear | 2 MHz |
| Stanford (N=24) | GE Healthcare Logiq E9 | Curved linear | 3-4 MHz |

**Table S 2 Breakdown of CEUS LI-RADS by different types of lesion**

| **Diagnosis** | **CEUS LR-1** | **CEUS LR-2** | **CEUS LR-3** | **CEUS LR-4** | **CEUS LR -5** | **CEUS LR-M** |
| --- | --- | --- | --- | --- | --- | --- |
| **Benign** | 1  (1%) | 1  (1%) | 7  (8%) | 4  (5%) | 0  (0%) | 0  (0%) |
| **HCC** | 0  (0%) | 0  (0%) | 11 (13%) | 15 (17%) | 42  (48%) | 3  (3%) |
| **ICC** | 0  (0%) | 0  (0%) | 0  (0%) | 0  (0%) | 0  (0%) | 3  (3%) |

**Table S 3 Breakdown of liver disease etiology by different type of lesions**

| **Diagnosis** | **ALD** | **Hep B** | **Hep C** | **NASH** |
| --- | --- | --- | --- | --- |
| **Benign** | 7  (8%) | 1  (1%) | 0  (0%) | 5  (6%) |
| **HCC** | 24  (28%) | 3  (3%) | 30 (34%) | 14 (16%) |
| **ICC** | 2  (2%) | 0  (0%) | 0  (0%) | 1  (1%) |

**Table S 4 Presence of cirrhosis for different types of lesion**

| **Diagnosis** | **No** | **Yes (biopsy confirmed)** |
| --- | --- | --- |
| **Benign** | 0 | 13 (0) |
| **HCC** | 5 | 66 (10) |
| **ICC** | 0 | 3 (0) |


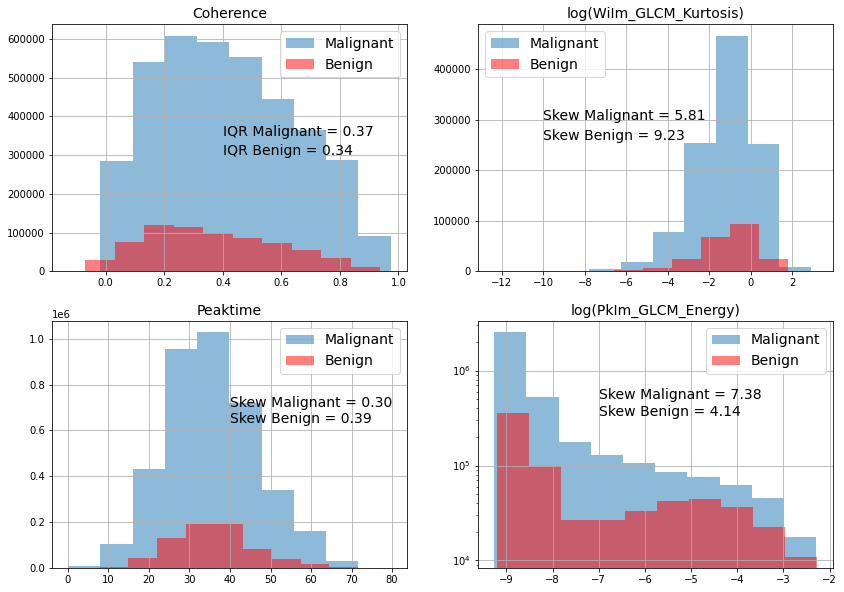


**Fig S 1** Histograms of the four most important features: the spatiotemporal features Coherence and Peaktime, and the texture features WiIm_Global_Kurtosis and PkIm_GLCM_Energy. For the texture features, the prefixes ‘WiIm’ and ‘PkIm’ indicate features extracted at wash-in and peak enhancement, respectively. Histograms have been obtained by pooling together all values in the ROI for all lesions. In each plot, the values of the most relevant summary statistic for the depicted feature are given as text for the malignant and benign samples, where IQR and Skew indicate the interquartile range and the skewness, respectively. To facilitate visualization, the feature WiIm_Global_Kurtosis is plotted in log scale on the x-axis, while the feature PkIm_GLCM_Energy is plotted in log scale on both axis.


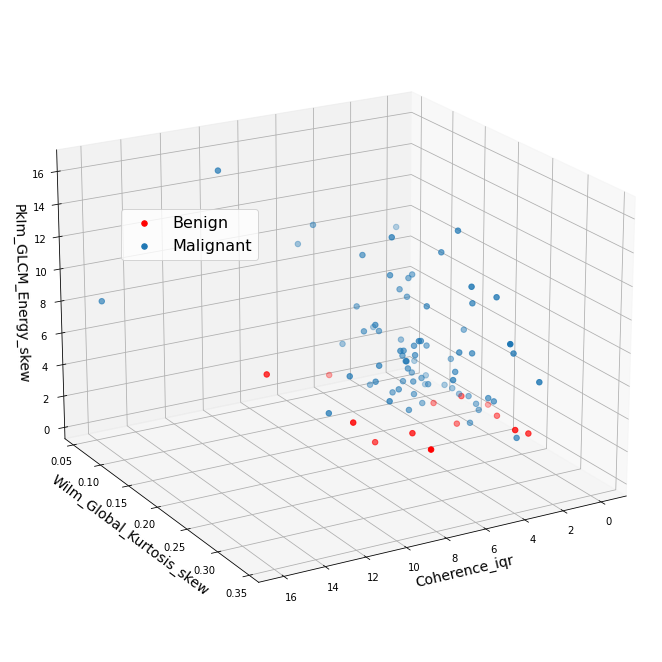


**Fig S 2** Scatter plot of the three most import features: Coherence_iqr, WiIm_Global_Kurtosis_skew and PkIm_GLCM_Energy_skew. For each point, the corresponding label is indicated in red for benign cases, and in blue for malignant cases. For each feature, the suffixes ‘iqr’ and ‘skew’, indicate the summary statistics interquartile range and skewness, respectively. For the texture features, the prefixes ‘WiIm’ and ‘PkIm’ indicate features extracted at wash-in and peak enhancement, respectively.
